# Growth Arrest-Specific Gene 6 Administration Ameliorates Sepsis-Induced Organ Damage in Mice and Reduces ROS Formation In Vitro

**DOI:** 10.3390/cells10030602

**Published:** 2021-03-09

**Authors:** Livia Salmi, Francesco Gavelli, Filippo Patrucco, Mattia Bellan, Pier Paolo Sainaghi, Gian Carlo Avanzi, Luigi Mario Castello

**Affiliations:** Department of Translational Medicine, University of Piemonte Orientale, 28100 Novara, Italy; livia.salmi@uniupo.it (L.S.); francesco.gavelli@uniupo.it (F.G.); filippo_patrucco@hotmail.it (F.P.); mattia.bellan@med.uniupo.it (M.B.); pierpaolo.sainaghi@med.uniupo.it (P.P.S.); giancarlo.avanzi@uniupo.it (G.C.A.)

**Keywords:** sepsis, Gas6, TAM receptors, organ damage

## Abstract

Sepsis is a widespread life-threatening disease, with a high mortality rate due to inflammation-induced multiorgan failure (MOF). Thus, new effective modulators of the immune response are urgently needed to ameliorate the outcome of septic patients. As growth arrest-specific gene 6 (Gas6)/Tyro3, Axl, MerTK (TAM) receptors signaling has shown immunomodulatory activity in sepsis, here we sought to determine whether Gas6 protein injection could mitigate MOF in a cecal slurry mouse model of sepsis. Mice, divided into different groups according to treatment—i.e., placebo (B), ampicillin (BA), Gas6 alone (BG), and ampicillin plus Gas6 (BAG)—were assessed for vitality, histopathology and cytokine expression profile as well as inducible nitric oxide synthase (iNOS), ALT and LDH levels. BAG-treated mice displayed milder kidney and lung damage and reduced levels of cytokine expression and iNOS in the lungs compared to BA-treated mice. Notably, BAG-treated mice showed lower LDH levels compared to controls. Lastly, BAG-treated cells of dendritic, endothelial or monocytic origin displayed reduced ROS formation and increased cell viability, with a marked upregulation of mitochondrial activity. Altogether, our findings indicate that combined treatment with Gas6 and antibiotics ameliorates sepsis-induced organ damage and reduces systemic LDH levels in mice, suggesting that Gas6 intravenous injection may be a viable therapeutic option in sepsis.

## 1. Introduction

Sepsis is a systemic response of the immune system arising from the spread of pathogenic agents (e.g., bacteria) in the body, which affects 50 million individuals every year worldwide. Alarmingly, ~20% of septic patients die worldwide because of sepsis complications and septic shock [1,2]. Besides bacterial resistance to antibiotics, the high mortality rate of sepsis is caused by severe organ dysfunction ultimately leading to multiple organ failure (MOF) [3,4]. Septic patients are indeed characterized by altered microcirculatory flow and microvascular dysfunction, which greatly contributes to MOF [5]. In addition, both septic patients [6] and murine models [7] of sepsis display metabolic reprogramming—with a shift toward aerobic glycolysis—and a positive association between mitochondrial dysfunction and sepsis severity. As a result of metabolism dysregulation, septic patients tend to have higher levels of plasma lactate, which serves as a biomarker of circulation impairment and prognostic stratification.

Our current understanding of the immune response to sepsis is controversial. Some investigators have proposed that the high mortality rates among septic patients may be ascribable to the aberrant and persistent activation of the immune system, while others argue that it is instead related to an acquired immunosuppression status [8,9]. However, there is general consensus on the need for early interventions aimed to restore and maintain tissue homeostasis in the affected organs, thereby reducing the risk of fatal outcome.

The most studied tyrosine kinase receptors that share Gas6 as ligand are Tyro3, Axl and MerTK (TAM); TAM receptors are differently expressed among tissues and organs, where they regulate tissue homeostasis [10]. Tyro-3 is mainly expressed in the central nervous system [11], while Axl and MerTK are ubiquitously expressed in immune sentinel cells, vascular endothelial cells, professional phagocytes of the immune, nervous and reproductive systems and several epithelial cell types [10]. In these settings, TAM signaling regulates multiple functions, such as the homeostatic balance and the inflammatory innate response of dendritic cells, macrophages and other immune cell types [12,13]. TAM receptors are activated upon binding to several ligands, among which growth arrest-specific 6 (Gas6) is a vitamin K-dependent protein normally circulating in the bloodstream found at increased plasma concentration during sepsis [14]. A common feature of Axl and MerTK is the presence of two fibronectin type III domains, making them susceptible to ADAM17- and lipopolysaccharide (LPS)-mediated shedding, which results in inactivation and downregulation of MerTK transcription [11].

More than ten years ago, the groups of Borgel and Gibot investigated for the first time the role of TAM and their ligands as biomarkers in septic patients, noticing a significant correlation between Gas6 and sepsis [15,16]. Analogously, Ekman and colleagues, some years later, observed a correlation between Gas6 and the degree of organ damage in sepsis [17]. Even though the immunomodulatory function of TAM receptors is well established in multiple systems [18,19], an important role of Gas6 in attenuating sepsis-induced tight junction injury and vascular endothelial hyperpermeability in vivo has only recently emerged [20]. Furthermore, the therapeutic role of Gas6 has also been suggested by two other research groups using mouse models of sepsis-induced kidney injury [21] and sepsis-induced lung injury [22], showing a reduction of urea nitrogen, creatinine, renal tissue apoptosis and a reduction in neutrophils-induced acute lung injury, respectively.

Thus, here we sought to determine the biological role of the Gas6/TAM axis in a mouse model of sepsis following broad-spectrum antibiotic administration. In particular, we aimed to determine whether the administration of full-length murine Gas6 protein combined with standard antibiotic therapy would ameliorate the widespread organ damage observed in septic mice.

## 2. Materials and Methods

### 2.1. Bacteria-Induced Septic Mouse Model

All animal studies were approved (Authorization number: 869/2016-PR) by the Animal Care and Use Committee of the Università del Piemonte Orientale (Novara, Italy). Male C57BL6 mice (*n* = 29) were used with weight ranging from 30 to 36 g and age from 13 to 26 weeks. Weight and age were used to match the different treatment groups. The eight treatment groups are shown in Table 1. For treatment, bacteria aliquots of cecal slurry stored at −80 °C were thawed at room temperature (RT), cultured in Luria broth (LB) medium for 30 min at 37 °C and 200 rpm, washed once and resuspended in phosphate-buffered saline (PBS).

Sepsis was induced by injecting intraperitoneally 10^8^ bacteria, whereas sterile PBS was injected in the control group. After ~16 h, 200 mg/kg ampicillin (Sigma Aldrich, St. Louis, MI, USA) was administered by tail vein (TV) injection as previously reported by Majhi et al. [23], while the control group received the same volume of PBS. Seven h after ampicillin injection, 5 µg of recombinant murine Gas6 (full-length protein; R&D systems, Minneapolis, MN, USA) and the same amount of PBS in control groups were tail vein injected in each mouse. Supplementary Figure S1 shows a schematic representation of the treatment timeline. Mice were monitored daily and fed with wet food to maintain hydration, and cages were warmed using heating pads to avoid hypothermia. Approximately 40 h after sepsis induction, mice were deeply anesthetized (isoflurane 1.5–2% O_2_), blood samples collected by intracardiac puncture and organs harvested for subsequent analysis. Due to difficulties in evaluating clinical parameter in mice, such as blood pressure, pale skin etc., a vitality score was set up and mice were evaluated in a double-blinded fashion. Each mouse was monitored and evaluated according to the following scoring system: 0 = dead mouse; 1 = living mouse, motionless with closed eyes, white ears and tachypneic; 2 = living mouse, slow movements, opened eyes, pink ears and normopneic; and 3 = living mouse, normal movements and normal behaviors.

### 2.2. Cell Culture Models of Sepsis

Monocytic Raw264.7 (ATCC^®^, Manassas, VI, USA, US-TIB-71™) cells were cultured in Dulbecco’s Modified Eagle’s Medium (DMEM) (Thermo Fisher Scientific, Waltham, MS, USA) in the presence of 10% fetal bovine serum (FBS), 2 mM glutamine (Sigma Aldrich, Darmstadt, Germany) and antibiotic solution (Sigma Aldrich, Darmstadt, Germany). Immature dendritic JAWSII cells (ATCC^®^ CRL-11904™) were maintained in Iscove’s Modified Dulbecco’s Medium (IMDM) (Thermo Fisher Scientific, Waltham, MA, USA) in the presence of 20% FBS 4 mM glutamine, 1 mM sodium pyruvate and antibiotic solution (Sigma Aldrich). Pancreatic islet endothelial MS1 cells (MILE SVEN 1) (ATCC^®^ CRL-2279™) were cultured in DMEM with 5% FBS and antibiotic solution.

### 2.3. Evaluation of Mitochondrial Activity

For cell treatment, bacteria were fixed with three volumes of paraformaldehyde (PFA) 4% in PBS for 3 h at 37 °C, washed twice, and resuspended in PBS to remove the excess of PFA before use. Since we used a normal cell culture room, we could not use live bacteria, which can proliferate and to contaminate other cell lines in the common incubator. Raw264.7 cells (30 × 10^3^), JAWSII cells (30 × 10^3^), and MS1 cells (20 × 10^3^), cultured in an antibiotic-free medium, were incubated for ~12 h in the presence or absence of 10^7^ PFA-fixed bacteria followed by 8-h treatment with ampicillin (0.1 mg/mL) (Sigma Aldrich, Darmstadt, Germany) and subsequent ~12-h incubation with Gas6 (100 ng/mL) (R&D Systems). The timeline of the various treatments is shown in Supplementary Figure S1.

Mitochondrial activity was assessed by MTT (3-[4,5-dimethylthiazol-2-yl]-2,5 diphenyl tetrazolium bromide; Sigma-Aldrich) assay. Cells were incubated for 90 min in the presence of 0.5 mg/mL MTT followed by formazan solubilization in dimethyl sulfoxide (DMSO). Since MTT assay does not discriminate proliferative effects, the quantification of mitochondrial activity was normalized to crystal violet (CV) staining, performed in parallel. Briefly, after treatments, cells were fixed in a 10% glutaraldehyde solution for 5 min at RT, washed twice in water, and stained for 15–20 min at RT with a CV solution (0.1% crystal violet + 20% methanol in water). Cells were washed extensively following staining, and CV was solubilized in 10% acetic acid in water. Both MTT and CV signals were measured at OD_570 nm_ using a Victor X microplate reader (PerkinElmer, Waltham, MS, USA). Mitochondrial activity was calculated as MTT OD_570 nm_/CV OD_570 nm_ ratio.

### 2.4. Quantification of Oxidative Stress

Raw264.7 (90 × 10^3^), JAWSII (90 × 10^3^) and MS1 cells (70 × 10^3^), plated on 15 mm round cover glass, were incubated for ~12 h in the presence or absence of 5 × 10^7^ PFA-fixed bacteria following 8-h treatment with ampicillin (0.1 mg/mL) (Sigma Aldrich, Darmstadt, Germany) and subsequent ~12 h incubation with Gas6 (100 ng/mL) (R&D Systems). Relative changes in intracellular reactive oxygen species (ROS) were measured using the oxidation-sensitive fluorogenic dye CellROX^®^ Green Reagent (Thermo Fisher Scientific, Waltham, MA, USA), and nuclei were stained with Hoechst (Thermo Fisher Scientific, Waltham, MA, USA), according to the manufacturer’s instructions, as previously described [24]. Dye-loaded cells were rinsed twice in Hank’s balanced salt solution (HBSS) and mounted on a glass slide using Mowiol (Sigma Aldrich, Darmstadt, Germany). Images were acquired using a fluorescence microscope (Leica DM 2500–Leica, Wetzlar, Germany) and analyzed (integrated density) with ImageJ software (NIH and LOCI, University of Wisconsin, Madison, WI, USA). To minimize photoactivation artifacts, cells fields were imaged under identical fluorescent conditions, using identical software settings.

### 2.5. RNA Extraction and Gene Expression Analysis

Organ samples (50–80 mg) were homogenized, and total RNA was extracted from each sample using TRIzol (Thermo Fisher Scientific, Waltham, MA, USA), according to the manufacturer’s instruction. RNA (1–3 μg) was retrotranscribed into cDNA with Applied Biosystems™ High-Capacity cDNA Reverse Transcription Kit. Gene expression analysis was performed using primers for Sybr Green assay or TaqMan Probes (Supplementary Table S1) and relative expression of candidate genes are relative to β-actin, which was use as housekeeping gene.

### 2.6. Histochemistry and Immunofluorescence

Formalin-fixed paraffin-embedded organs were processed for morphological (H&E) and immunohistochemical (MPO) staining. For MPO staining, spleen sections were incubated with anti-myeloperoxidase rabbit polyclonal antibody (Cell Marque™, Rocklin, CA, USA) diluted 1:200. Histological images were acquired using a Pannoramic MIDI II (3DHISTECH™, Budapest, Hungary). Images were analyzed using QuPath software (open-source digital pathology, University of Edinburgh, Edinburgh, UK). With this method, we also evaluated the ischemic areas in the kidney section of each mouse. Mouse tissues were fixed in 4% PFA for 2 h at 4 °C, equilibrated in sucrose, and embedded in cryostat embedding medium (Bio-Optica, Milano, Italy). Six- to 7-μm-thick Killik-embedded mouse organs sections were fixed in 4% PFA for 10 min at 4 °C and permeabilized with ice-cold 0.5% Triton X-100 in PBS for 10 min. Samples were then incubated for 2 h at RT with 1:200 dilutions of the following antibodies: Tyr691 phospho-Axl (pAxl) (rabbit polyclonal; Invitrogen, Carlsbad, CA, USA, PA5–39729, previously validated [25]) and Tyr681, Tyr749 phosphor-MerTK (pMer) (rabbit polyclonal; Abcam, Cambridge, UK, ab192649, previously validated [26]). Hematopoietic cells were detected using a rat anti-mouse CD45 (clone IBL-5/25; Immunotools, Friesoythe, Germany) diluted 1:200, for 2 h at RT. After washing in PBS, Alexa Fluor^®^488- or 546-conjugated goat anti-rabbit, anti-rat IgGs (1:500, Molecular Probes) were added for 1 h. Nuclei were stained with 4′,6-diamidino-2-phenylindole (DAPI; Sigma Aldrich, Darmstadt, Germany) or TO-PRO-3 (Thermo Fisher Scientific, Waltham, MA, USA). Images were acquired using fluorescence (Leica DM 2500) or confocal (Leica TCS SP2) microscope.

### 2.7. Statistical Analysis

Data are expressed as mean ± SD or median [IQR] as appropriate. Normality of data distribution was evaluated by the Kolmogorov–Smirnov test. One-way ANOVA, two-way ANOVA and *t*-test for independent samples were performed to analyzed parametric data, while the Kruskal–Wallis test was performed for non-parametric data, as appropriate. Gene expression analysis for cytokines (IL1-β, IL6, IL10, TGF-β, TNF-α) was generated as follows: the mean differences of each gene were calculated by multiple *t*-tests of relative expression (between P group and treatment conditions). The comparison among groups was then performed by ANOVA Tukey’s multiple test. Statistical significance was set at a *p* value < 0.05.

Statistical analysis was performed with both StataIC software (Version 15, StataCorp LLC, College Station, TX, USA) and GraphPad Prism 6 software (GraphPad Software, San Diego, CA, USA). Graphs were created using GraphPad Prism 6 software.

## 3. Results

### 3.1. Groups of Mice Divided for Treatments and Timeline Treatments

Animals were divided in eight groups of treatments: group 5 (*n* = 3 mice), 6 (*n* = 3), 7 (*n* = 3) and 8 (*n* = 3) were control groups, whereas group 1 (*n* = 4), 2 (*n* = 4), 3 (*n* = 4) and 4 (*n* = 5) were septic mice treated differently with antibiotic standard therapy and/or growth arrest-specific 6 (Gas6). All mice were matched by their weight. 10^8^ bacteria were intraperitoneally injected (control groups were treated with PBS intraperitoneal injection); Ampicillin was tail-vein injected as broad-spectrum antibiotic (200 mg/kg); 5 µg of Gas6 was tail-vein injected (Table 1).

### 3.2. Gas6 Administration Ameliorates Vitality and Organ Damage of Septic Mice

**Figure 1:**
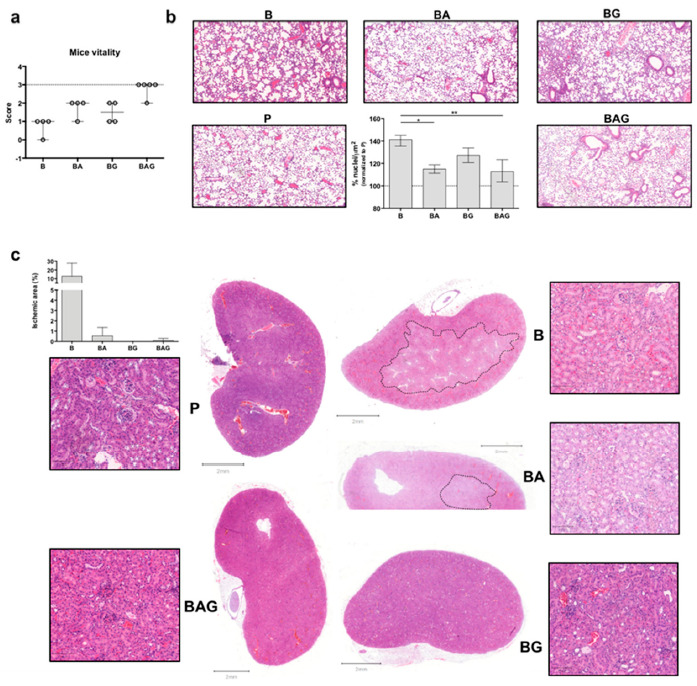
Mouse vitality and macroscopic organ evaluation. (**a**) Mice were classified according to the following vitality scheme. 0 = live mouse, motionless with closed eyes, white ears and tachypneic, 2 = live mouse, slow movements opened eyes, pink ears and normopneic; 3 = live mouse, normal movements/normal behaviors. The dotted line corresponds to the score (3) of control mice (P). (**b**) H&E staining of lung sections showing an increased number of infiltrating cells in B (septic) mice compare to the other groups. The histogram shows the percentage (average ± SD) of infiltrating cells in the lungs of all treated mice. Values are calculated as a number of nuclei per area (µm^2^) normalized to P (control groups) average (%). (**c**) H&E staining of kidney sections—whole sections and magnified areas (lateral squares; 100 µm magnification/area). The dotted lines show ischemic areas found in kidney sections from B and BA mice. The histogram represents the ischemic area (µm^2^)/whole area (µm^2^) as percentage (%). For all the analyses septic mice (B = bacteria, septic mice *n* = 4), septic-treated mice (BA = bacteria + ampicillin, *n* = 4; BG = bacteria + Gas6, *n* = 4; BAG = bacteria + ampicillin + Gas6, *n* = 5) and control mice (P = PBS, *n* = 4) were included. Values are represented as average ± SD. * *p* < 0.05, ** *p* < 0.01.

To avoid bias in monitoring the clinical parameters in septic mice, we devised a mouse vitality score (see Material and Methods) that was used in a double-blind fashion. While healthy mice—i.e., mice injected with PBS (group P, control mice, *n* = 4)—, obtained a score of 3 (Figure 1a), mice injected with bacteria to induce sepsis (group B, *n* = 4) had a score of 0–1. Septic mice treated with broad spectrum antibiotic (group BA, *n* = 4), the current standard therapy for septic patients, obtained a score of 1–2, similar to that of septic mice treated with Gas6 (group BG, *n* = 4). Interestingly, 4 out of 5 septic mice receiving both antibiotic and Gas6 (group BAG, *n* = 5) obtained a mean score of 2.8 (±0.4), almost identical to that of healthy mice (3) (Figure 1a).

As septic patients frequently develop acute respiratory distress syndrome (ARDS) [27] and acute kidney injury (AKI) [28], we next evaluated septic vs. treated lung and kidney tissues by histological analysis. While we observed a dramatic increase in the number of infiltrating cells in the alveoli of mice from group B, BA and BAG mice showed a significant decrease in the number of total cells in pulmonary tissue (Figure 1b), consistent with a reduction of inflammation. Furthermore, septic kidneys from B mice showed an extended ischemic area (Figure 1c) with tubule edema. These ischemic areas with pale staining and unstructured glomeruli were also visible in the kidneys from BA mice, while septic mice receiving Gas6 and Gas6 plus antibiotic (BG and BAG, respectively) showed a better histological outcome, similar to that seen in the kidneys from control mice (P) (Figure 1c). The histogram shows the percentage of ischemic kidney area if present: specifically, 3 mice out of 4 in group B, one mouse out of 4 in group BA and one mouse out of 5 in group BAG developed renal ischemia (Figure 1c, histogram). No histological differences were observed in liver (except of 1 septic mouse (B) (Supplementary Figure S3a,b). Thus, the administration of full-length Gas6 protein in association with antibiotic appears to reduce organ damage in the kidney and lung in septic mice.

### 3.3. Gas6 Restores Physiological Homeostasis in Septic Mice

**Figure 2:**
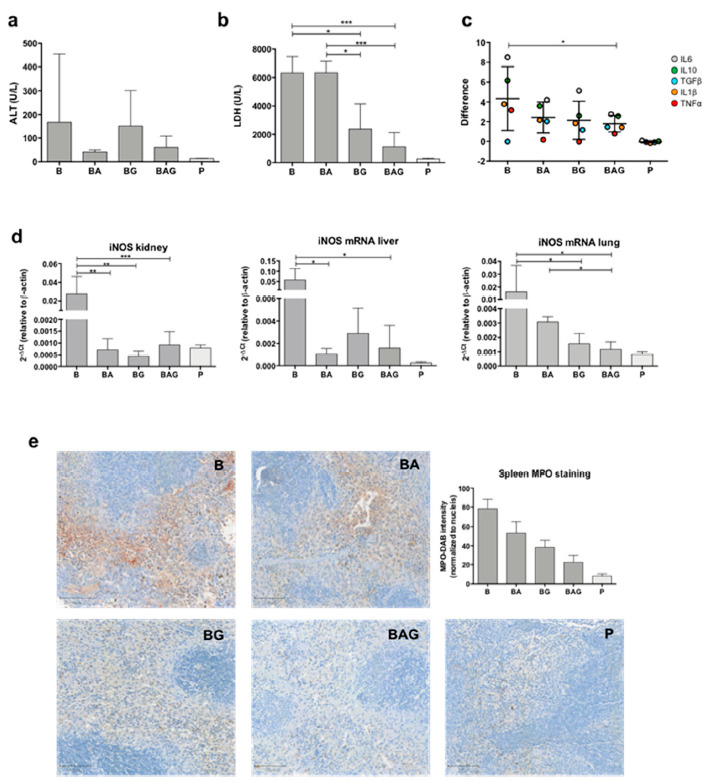
Mouse organ functionality. (**a**) Alanine aminotransferase (ALT) levels (U/L) from all treatment groups and control P mice, grouped according to Table 1. (**b**) Lactate dehydrogenase (LDH) levels (U/L) in the different treatment groups. (**c**) Difference distribution between the control group (dotted line) and treatment groups (i.e., B, BA, BG and BAG) of logarithmic cytokine mean (± SD) from all organs analyzed (i.e., liver, kidney and lung). The pro- and anti-inflammatory cytokines analyzed were interleukin-1β (IL-1β) (orange dots), IL-6 (grey dots), IL-10 (green dots), TGF-β1 (blue dots) and TNF-α (red dots). Data comparison was performed through the two-way ANOVA (**d**) Inducible nitric oxide synthase (iNOS) mRNA expression in the kidney, liver and lung from all treatment group and control P group(**e**) Representative images and quantification (as MPO intensity/number of nuclei) of myeloperoxidase staining (brown color) in spleen samples from treated mice. For all analysis septic mice (B = bacteria, septic mice *n* = 4), septic-treated mice (BA = bacteria + ampicillin, *n* = 4; BG = bacteria + Gas6, *n* = 4; BAG = bacteria + ampicillin + Gas6, *n* = 5) and control mice (*p* = PBS, *n* = 4) were included. Values are represented as average ± SD. * *p* < 0.05, ** *p* < 0.01, *** *p* < 0.005.

Liver failure is one of the components of sepsis-related MOF. By measuring plasma ALT levels of each mouse, we found high variability but not significant differences among mice groups (Figure 2a). This finding allowed us to rule out liver toxicity induced by Gas6 and antibiotics, both metabolized by the liver.

During sepsis and septic shock, organ damage and cell death increase the concentration of lactate dehydrogenase (LDH) in the bloodstream. Of note, a recent study has shown that LDH concentration is associated with 28-day mortality in patients with sepsis [29]. Consistent with an opposing role of Gas6 in sepsis-induced MOF, we recorded a considerable decrease in plasma LDH levels in mice treated with Gas6 alone or in combination with ampicillin compared to standard antibiotic therapy (Figure 2b) (BG = 2368 U/L, ±1776, *p* = 0.011; BAG = 1115 U/L, ±1001; BA = 6328 U/L, ±821.8; *p* = 0.0007, respectively).

As sepsis-induced disruption of tissue homeostasis leads to aberrant regulation of both anti- and pro-inflammatory cytokines (CKs), we sought to determine the expression of IL-1β, IL-6, IL-10, TGF-β1, and TNFα, the main drivers of tissue inflammation. To this end, CK mRNA expression in the kidneys, livers and lungs from B, BA, BG and BAG mice was assessed by real-time qPCR analysis and compared to that of control animals. Figure 2c shows the difference distribution between the control group (P) and the various treatment groups (i.e., B, BA, BG and BAG) in terms of CK expression levels, which was calculated by pooling together the results obtained from all organs analyzed (i.e., liver, kidney and lung) according to the treatment group (B = 4.3 ± 3.2; BA = 2.4 ± 1.6; BG = 2.1 ± 1.9; BAG = 1.8 ± 0.8; mean difference ± SE). The distribution of systemic CKs was significantly different between B and BAG mice (*p* = 0.037). In particular, B mice showed high variability in the expression of all CKs analyzed, which was associated with homeostasis disruption. In contrast, mice receiving antibiotic therapy (BA) or Gas6 (BG) showed a reduction in CKs expression variability. Finally, BAG mice showed a statistically significant difference in CK expression, with an overall improvement of systemic homeostasis, as judged by a more homogeneous CK distribution similar to that of control mice (Figure 2c).

ROS are involved in tissue homeostasis disruption, especially in sepsis [30]. Moreover, overproduction of nitric oxide (NO) due to inducible NO synthase (iNOS) activity has been associated with harmful effects, such as general vasodilatation and vasoplegia [31]. Thus, we next investigated iNOS expression in the kidneys, livers and lungs of treated and control mice. As expected, B mice showed a significant upregulation of iNOS mRNA in all three organs compared to control mice (Figure 2d). By contrast, we found lower iNOS mRNA levels in septic mice treated with antibiotics and/or Gas6 compared to B mice (Figure 2d). Interestingly, iNOS concentration was significantly downregulated in the lungs of BAG vs. BA mice (Figure 2d)—0.0012 (2^−Δct^) ± 0.0005 vs. 0.0030 (2^−Δct^) ± 0.0004, *p* = 0.041, respectively. Lastly, we detected overexpression of myeloperoxidase (MPO) in splenic red pulp from septic mice (Figure 2e), while septic mice treated with antibiotic and/or Gas6 showed decreased MPO expression, with BAG mice resembling control animals in terms of MPO staining (Figure 2e). Quantification of MPO intensity staining, normalized by number of nuclei, was assessed as previously described [32] for septic (B, *n* = 4), septic-treated (BA, *n* = 4; BG, *n* = 4; BAG, *n* = 5) and control mice (P, *n* = 4) and shown in the histogram in Figure 2e. The differences among groups were statistically significant (*p* < 0.001).

### 3.4. Gas6 Decreases ROS Production and May Improve Mitochondrial Function

Since we observed a significant decrease in systemic LDH levels and iNOS expression, we decided to assess ROS formation in vitro. For this purpose, we used the Raw246.7, JAWSII and MS1 cell lines as they recapitulate the most frequently targeted cell lineages during sepsis (i.e., monocytes/macrophages, dendritic cells and endothelial cells, respectively) [3]. For these in vitro experiments, we employed the same timeline as that used for the in vivo experiments. Of note, these cells were exposed to PFA-inactivated bacteria to induce sepsis (Supplementary Figure S1).

**Figure 3:**
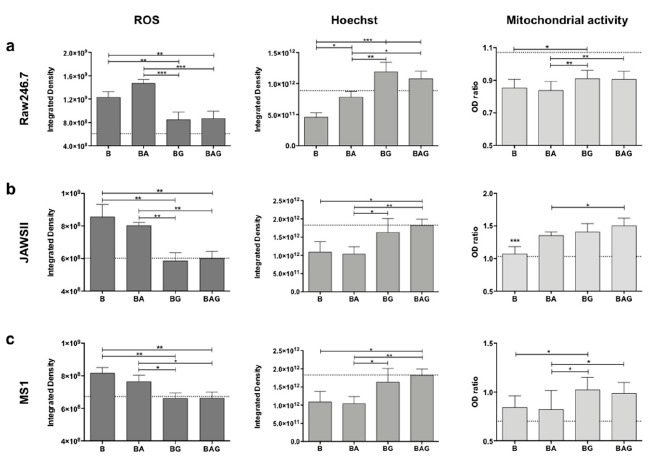
Reactive oxygen species (ROS) production, cell viability and mitochondrial activity in Raw246.7 (**a**), JAWSII (**b**) and MS1 (**c**) cells. The histograms represent mean values ± SD. The dotted lines represent the mean values of control cells. B = PFA-inactivated (PFAi) bacteria; BA = PFAi bacteria + ampicillin; BG = PFAi bacteria + Gas6; BAG = PFAi bacteria+ ampicillin + Gas6. * *p* < 0.05, ** *p* < 0.01, *** *p* < 0.005.

ROS production, measured as integrated density with ImageJ software, was significantly upregulated in all three cell lines treated with fixed bacteria (B) but was not reduced by ampicillin treatment (BA) (Figure 3a–c). By contrast, ROS levels were significantly reduced in all three cell lines following treatment with Gas6 (BG and BAG) (Figure 3a–c), with both JAWSII and MS1 cells displaying ROS levels similar to those of control cells (P). Furthermore, a statistically significant reduction was observed in all three BAG- vs. BA-treated cell lines (Figure 3a–c). Moreover, Hoechst staining revealed a much higher number of dead cells in B and BA vs. BAG and BG mice (Figure 3a–c).

Finally, we assessed whether Gas6-treated cells displayed an increased metabolism during infection (Figure 3a–c). BAG-treated cells showed a much stronger reduction in ROS production compared to that of BA-treated cells in all three cell lines, suggesting that the addition of Gas6 to the standard treatment (antibiotics) may improve mitochondrial function and cell metabolism.

### 3.5. Gas6 Differently Activates Axl and MerTK in Hematopoietic and Parenchymal Cells in Sepsis

**Figure 4:**
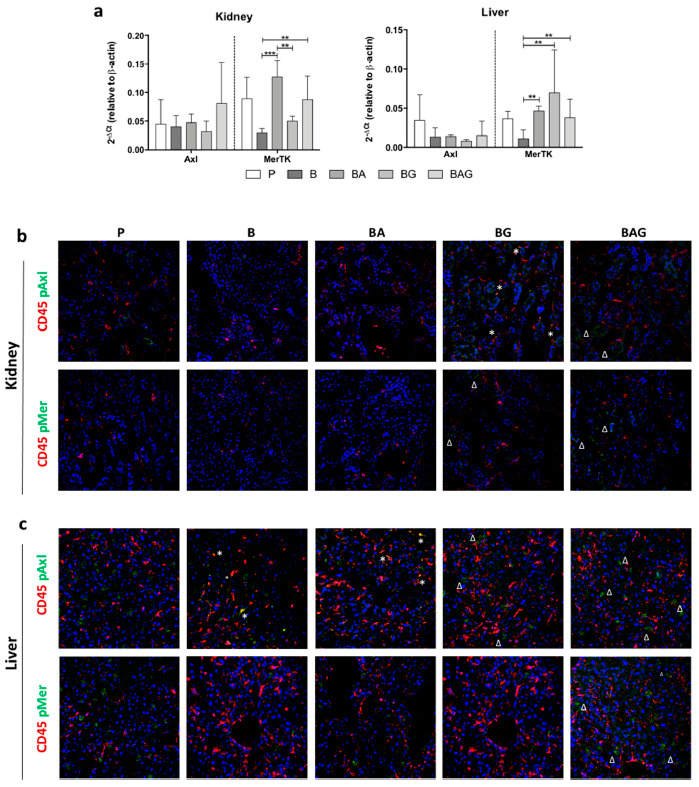
MerTK and Axl expression and activation in the liver and kidney. (**a**) MerTK and Axl mRNA expression levels in the kidney (upper panel) and liver (lower panel) of mice from the treatment groups described in the legend to Figure 1 (i.e., P, B, BA, BG and BAG). The bar graphs represent mean ± SD. ** *p* < 0.01, *** *p* < 0.005. Immunofluorescence on kidney (**b**) and liver (**c**) sections showing protein expression of CD45 (red) and phospho-Axl (pAxl) or phospho-MerTK (pMer) (green). The white asterisks (*) indicate pMer/pAxl and CD45 co-staining, whereas the white deltas (Δ) indicates pAxl/pMer localized in CD45-negative cells. For all analysis septic mice (B = bacteria, septic mice *n* = 4), septic-treated mice (BA = bacteria + ampicillin, *n* = 4; BG = bacteria + Gas6, *n* = 4; BAG = bacteria + ampicillin + Gas6, *n* = 5) and control mice (*p* = PBS, *n* = 4) were included.

As circulating Gas6 has several target receptors scattered in different tissues and organs, we focused our attention on the two most important receptors expressed outside the central nervous system, namely Axl and MerTK. In particular, we asked whether improved kidney damage following Gas6 administration was associated to the activation of Axl, MerTK or both. Furthermore, we decided to evaluate the effects of Ga6 administration also on the liver, even though in this case, we had not observed significant changes in terms of organ damage. At the mRNA level, we found no significant changes in Axl expression in the liver and kidney across all treatment groups (Figure 4a). Although we did not observe significant differences in MerTK expression between control, BA and BAG mice, MerTK was significantly upregulated in the kidney from BA and BAG mice compared to B mice, and in the liver from BG mice compared to B mice (Figure 4a).

Both Axl and MerTK are tyrosine kinases whose activity depends not only on their expression but also on their activation in response to tyrosine phosphorylation. Thus, we next investigated the phosphorylation status of these two receptors in kidney and liver tissue sections by immunofluorescence using specific anti-phospho antibodies (pAxl; pMer). In addition, these tissue sections were co-stained with an antibody directed against CD45, a marker of hematopoietic cells. We reasoned that co-localization of pMer and pAxl with CD45 would have allowed us to determine whether these receptors exerted their protective role by immune hematopoietic cells or resident parenchymal cells.

In control kidney cells, we could only detect a slight positivity for pAxl, which was never colocalized with CD45 staining (Figure 4b). Furthermore, we found undetectable pAxl and pMer levels in both B and BA mice. In contrast, BG and BAG mice displayed phosphorylation of both Axl and MerTK receptors in kidney parenchymal cells, but we observed pAxl-CD45 co-localization only in BG-derived tissue (Figure 4b).

Unlike the kidney, the liver displayed strong phosphorylation of Axl and MerTK in parenchymal cells from the control group (P) and from BG and BAG mice, with BG mice showing less activation compared to P and BAG mice (Figure 4c). In contrast to the kidney, the liver of B and BA mice showed extensive pAxl-CD45 co-localization (Figure 4c). Taken together, these observations suggest that Gas6 administration ameliorates sepsis-driven organ damage and that this effect is likely mediated by parenchymal cells rather than hematopoietic cells.

## 4. Discussion

In this study, we investigated the possible therapeutic efficacy of Gas6 administration following antibiotic therapy in a murine model of sepsis. We decided to induce sepsis using the cecal slurry model instead of cecal ligation and puncture (CLP) since the cecal slurry methodology overcomes CLP-related variability and avoids performing a thoroughly invasive surgery, which can constitute major biases in sepsis models [33].

Our results indicate that intravenous Gas6 administration, alone or in association with antibiotics, can ameliorate the general health status of septic mice. Indeed, Gas6-treated septic mice showed improved vital signs (BG mice), and the combined treatment with antibiotics and Gas6 (BAG mice) further improved their condition, making them almost indistinguishable from their healthy counterparts. With the exception of one mouse, BAG mice were fairly active and characterized by normal respiratory rate and regular vascular blood supply, as judged by the pink color of their ears. Conversely, septic mice treated with antibiotics alone (BA mice) showed a more compromised health status, with signs of disease severity such as tachypnea, closed eyes, white ears and reduced movements.

These clinical findings were confirmed at the histological level. In the first 36 h, we found a substantial reduction of inflammatory activity in both the kidneys and lungs from BAG mice. In our opinion, this is a very promising finding, since sepsis-related lung damage, leading to ARDS, greatly increases the morbidity and mortality rate of septic patients [34].

In our study, both BA and BAG mice showed a significant decrease in the number of total cells found in the alveolar space, as calculated by QuPath software and reported in Figure 1b, thus suggesting a reduction of infiltrating cells. These data suggest that Gas6 injection does not hamper the therapeutic efficacy of antibiotics, which is consistent with the fact that Gas6 administration improves ARDS conditions in CLP-induced sepsis [22].

With regard to the involvement of the kidneys during sepsis, a recent study has shown that early reversible AKI within 24 h is associated with improved survival, whereas long-term survival of patients with sepsis-associated AKI is strongly related to recovery status at hospital discharge [35]. Although we did not measure serum creatinine, commonly used to monitor renal function, our histological examination showed that the renal structure observed in BAG mice was comparable to that of control mice, while BA mice displayed a de-structured kidney, with ischemic areas and tubular damage similar to those seen in septic mice. Thus, our findings appear to indicate a protective role of Gas6 also in sepsis-induced kidney injury.

Even though we observed an alteration in ALT levels following Gas6 injection, particularly evident in BG mice, such increase was not statistically significant—additional analyses are needed to fully understand the meaning of this response. On the other hand, it was truly surprising to observe a drastic and significant reduction in plasma LDH levels in BAG vs. BA mice, with the latter group showing values comparable to those of septic mice. It is well known that the reduction of lactate and LDH levels is crucial to achieve a better disease outcome. Indeed, lactate elevation has been classically attributed to global hypoperfusion in the setting of hemorrhagic shock, but it is well known that in sepsis additional mechanisms, such as accelerated glycolysis, may cause hyperlactatemia [36,37]. Additionally, it has been recently observed that high serum LDH levels are independently associated with 28-day mortality in patients with sepsis [29]. In this regard, accelerated glycolysis can be strictly related to mitochondrial dysfunction and oxidative stress induction [38,39].

Another important effect of Gas6 treatment is a tendency to rebalance the levels of pro- and anti-inflammatory cytokine expression in the different organs, particularly evident in BAG vs. septic mice. This finding is consistent with the role of the Gas6/TAM axis in the regulation of the inflammatory response during sepsis [10,40].

In line with this observation, we also noticed a significant decrease in ROS production in our in vitro experiments, accompanied by increased cell viability. Both ROS production and altered microvascular mechanisms are partially linked to NO and iNOS production. Heemskerk and colleagues reported that the use of selective iNOS inhibitors attenuates sepsis-induced renal dysfunction and improves survival in animals [41]. More recently, iNOS knockout mice showed improved survival rate against CLP-induced sepsis and were less likely to develop ARDS [42]. In our study, BAG mice showed significant iNOS downregulation and, especially in the lungs from BAG mice, iNOS levels were significantly lower compared to mice receiving antibiotics. Likewise, MPO levels in the spleen from BAG mice were comparable to those seen in healthy control mice, while BA mice showed a remarkably higher MPO expression. It is noteworthy that the restoration of MPO expression in the spleen of BAG mice is in line with of the findings by Schrijver et al., showing that plasma MPO levels can be used as a marker to diagnose and predict mortality in septic patients [43]. Furthermore, Theeß and colleagues observed that malaria-infected MPO-deficient mice did not show increased parasite loads and, unexpectedly, were able to clear the infection more rapidly than wild-type mice [44]. It is well known that MPO is a “wake-up call” of inflammation or local tissue damage and, from a systemic point of view, elevated MPO levels in circulation are associated with increased oxidative stress [45] and can predict adverse clinical outcomes in critical pathologies, especially in cardiovascular diseases [46,47].

In our experiments, we observed a concomitant reduction of both MPO/iNOS levels in vivo and ROS formation in vitro when Gas6 was added. To date, it is consolidated that among Gas6 biological effects, migration, cell growth, and cell survival can be listed [14,48]. Thus, from our preliminary data, we propose that Gas6 may play a role in preventing tissue damage exacerbation induced by MPO. Further analyses are required to verify this hypothesis.

Lastly, we focused our attention on the two Gas6 receptors Axl and MerTK, which are ubiquitously expressed and play an important role in inflammatory responses. In particular, we found MerTK mRNA expression to be modulated in the liver and kidney in response to the various treatments, whereas no statistically significant variation was observed in Axl mRNA expression. However, since the activity of these two tyrosine kinase receptors mainly depends on tyrosine phosphorylation, we evaluated their phosphorylation status in our experimental model. Intriguingly, in kidney tissues from B and BA mice, we detected pAxl expression in CD45-positive cells, but such colocalization was never found in tissues from BG and BAG mice as well as control mice, indicating that the Gas6/TAM axis on myeloid cells is not directly involved in the response following Gas6 administration. This finding is in line with the expression of Mer and Axl in mesangial and endothelial glomerular cells, previously reported [49]. In the liver, pMer did not colocalize with CD45-positive cells in any mouse group, but it was detected around small-caliber vessels, in agreement with previous data by Mukherjee SK and colleagues, showing MerTK expression in liver sinusoidal endothelial cells and hepatic stellate cells, but not hepatocytes [50]. Thus, systemic administration of Gas6 is able to induce a response against sepsis not only from myeloid cells, either resident or recruited from the bloodstream, as one would expect, but also from parenchymal cells.

This study has several limitations, since further analyses are clearly needed to better understand and characterize the role of Gas6 and its mechanisms in a complex disease, such as sepsis and sepsis-induced organ damage. Moreover, to better understand the biological role of iNOS in this setting, NO determination would be helpful to link in vitro and in vivo iNOS expression results. Again, the number of mice should be increased to obtain more robust data, evaluate long-term effects and investigate different Gas6 doses in order to understand the minimal effective dose to achieve protection. Notwithstanding, a single dose of Gas6 seems to have a strong beneficial effect in restoring homeostasis and limiting organ damage in septic mice, by reducing systemic LDH levels, decreasing ROS formation and intensifying mitochondrial activity. The fact that Gas6 is an endogenous molecule normally circulating in the body under physiological conditions makes this protein a particularly attractive therapeutic candidate for clinical development.

## Figures and Tables

**Table 1:** Group of mice divided for treatments. 108 bacteria intraperitoneally injected, 200 mg/kg tail vein injected and 5 µg growth arrest-specific 6 (Gas6) tail vein injected.

Treatment Group	Treatment(s)	Median Weight (g)	Number of Mice
1	Bacteria (B)	33	4
2	Bacteria + Ampicillin (BA)	33.5	4
3	Bacteria + Gas6 (BG)	33	4
4	Bacteria + Ampicillin + Gas6 (BAG)	33	5
5	Ampicillin (A)	34	3
6	Gas6 (G)	33	3
7	Ampicillin + Gas6 (AG)	33.5	3
8	PBS (P)	33.5	3

## Data Availability

All results are stored on a durable support; access to the data is reserved for the PI of the study. Data are available for consultation only with specific authorization from the PI.

## Supplementary Materials

The following are available online at https://www.mdpi.com/2073-4409/10/3/602/s1, Table S1: Primers sequence and TaqMan probes. Figure S1: Treatment timeline. Figure S2. Liver histology with hematoxylin-eosin (H&E) staining. Figure S3. Kidney (a) and lung (b) histology with hematoxylin-eosin (H&E) staining for control mice. Figure S4. Organ function of control mice. Figure S5. In vitro control treatments. Figure S6. Representative fluorescent images of reactive oxygen species (ROS). Figure S7. Relative expression (2^−ΔCt^) of cytokines measured in liver, kidney and lung tissues.
