# Supplementary material for: Growth Arrest-Specific Gene 6 Administration Ameliorates Sepsis-Induced Organ Damage in Mice and Reduces ROS Formation In Vitro
Source: Cells. 2021 Mar 9;10(3):602. doi: 10.3390/cells10030602 (PMC7998241; doi:10.3390/cells10030602)
Supplement: Supplementary file 1 [file cells-10-00602-s001.pdf]

cells MDPI

SUPPLEMENTAL MATERIAL

TableS1. Primers sequence and TaqMan probes.

Gene	Forward Primers	Reverse Primer
β-actin	5’ tgcctgacggccaggtcat 3’	5’ atctccttctgcatcctgtcgg 3’
Interleukin-1β	5’ gctgtcctgatgagagcatcc 3’	5’ catgagtcacagaggatgggctc 3’
Interleukin-6	5’ gctggtgacaaccacggccttc 3’	5’ ttctgcaagtgcatcatcgttgttc 3’
Interleukin-10	5’ agccttatcggaaatgatccagt 3’	5’ ggccttgtagacaccttggt 3’
TGF-β	5’ ctcccgtggcttctagtgc 3’	5’ gccttagtttggacaggatctg 3’
MerTK	5’ ctctggagtggaggcactg 3’	5’ atcttccagtctggggtggt 3’
Axl	5’ gaacttgccaggctcctactct 3’	5’ ggagttgacacaggtctgctca 3’
iNOS	5’ ccccgctactactccatcag 3’	5’ ccactgacacttcgcacaaa 3’
**Gene**	**TaqMan probe**	**GeneBank**
β-actin	Mm00607939_s1	AK07935.1
TNFα	Mm00443258_m1	AK153319.1

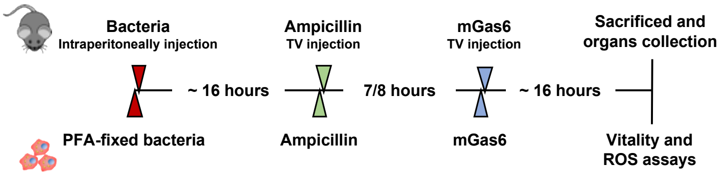

FigureS1. Timeline of treatments. Upper layer timeline in vivo and lower layer timeline of treatments in vitro.

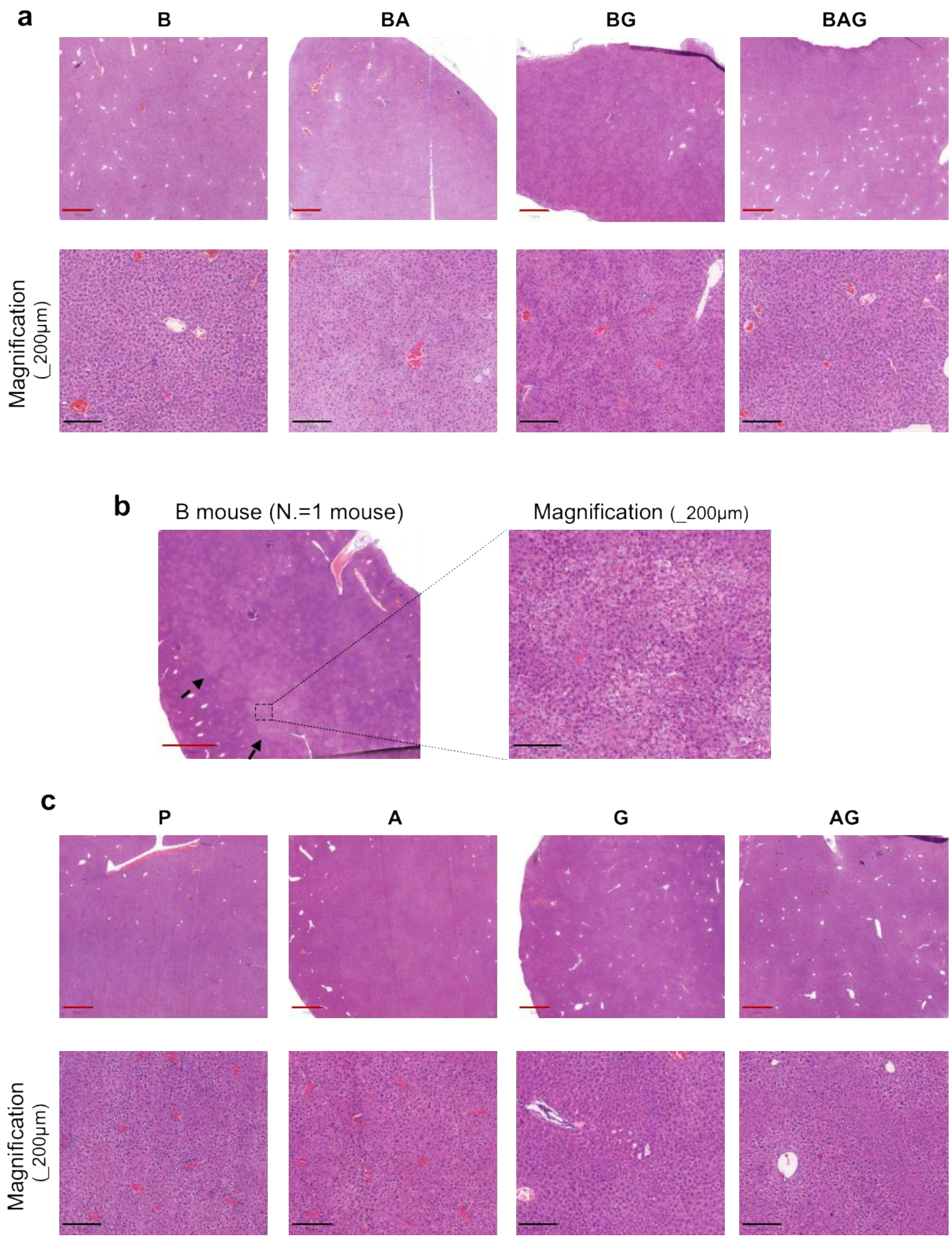

Figure S2. Liver histology with hematoxylin-eosin (H&E) staining. **a**) H&E liver staining of septic (B) and septic treated mice (BA, BG, BAG). Upper panels shown no magnified images (red lines = 1 mm), whereas in the lower panels are shown magnified areas (black bars = 200 µm). **b**) N.=1 (out of 4) septic mouse without treatment displayed ischemic area in liver (highlighted by black arrows in the left panels, red line = 2 mm, black line = 200 µm). c) H&E liver staining of control mice (P, A, G and AG) Upper panels shown no magnified images (red lines = 1 mm), whereas in the lower panels are shown magnified areas (black bars = 200 µm).

cells MDPI

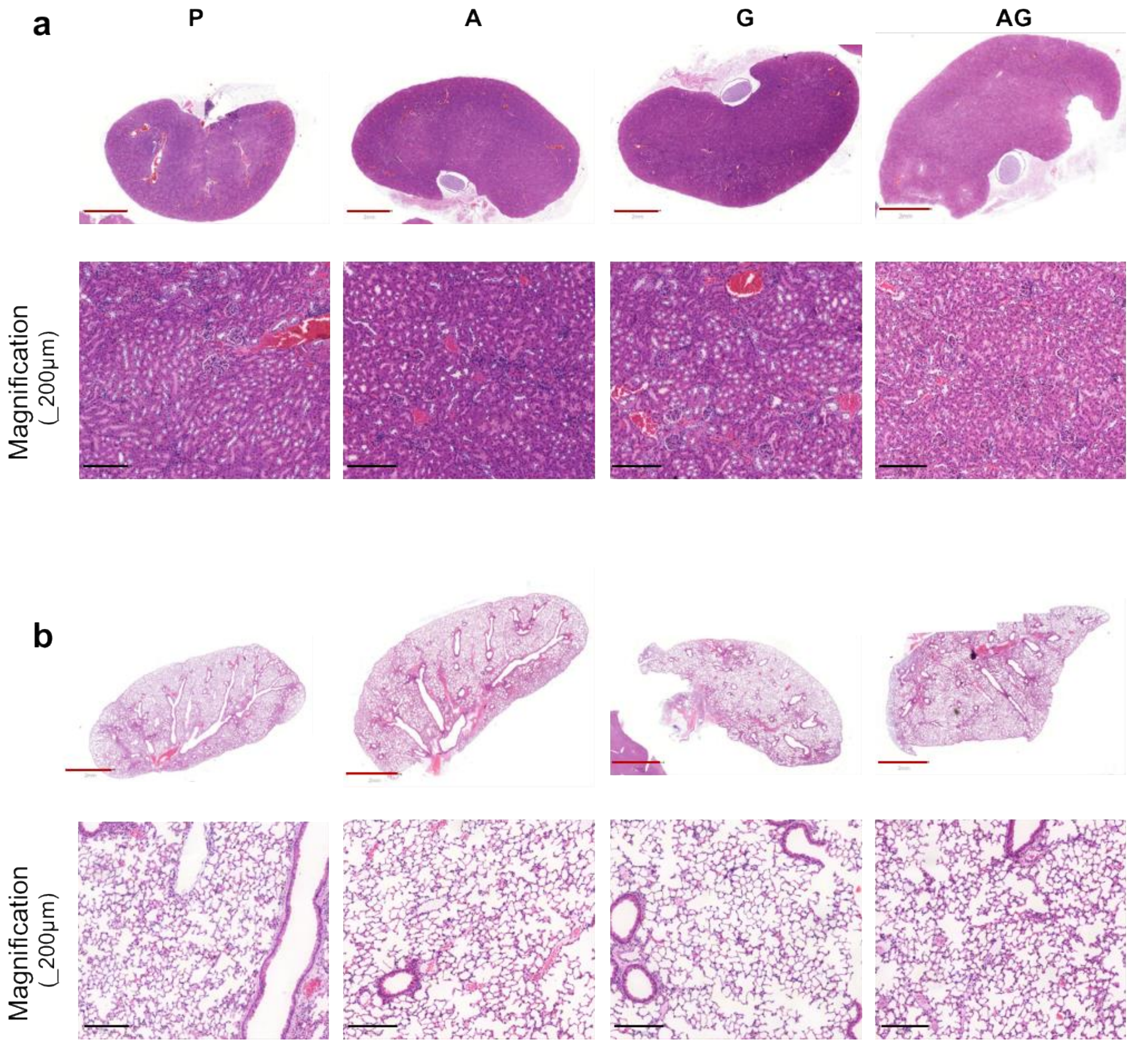

Figure S3. Kidney (a) and lung (b) histology with hematoxylin-eosin (H&E) staining for control mice (P, A, G and AG). In upper panels are present the whole organs, while in lower panels are shown magnification. Red lines = 2 mm, black lines = 200 µm.

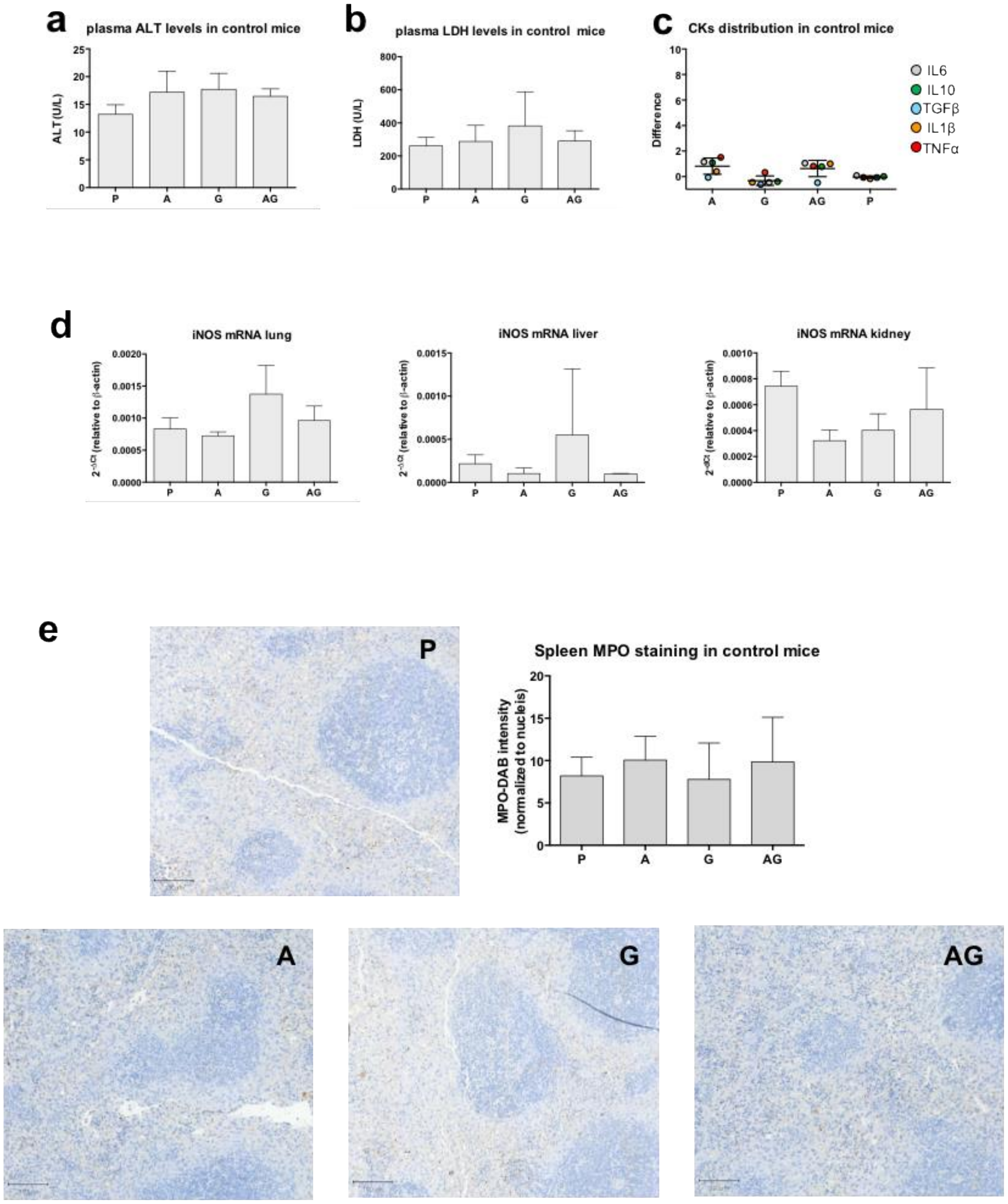

Figure S4. Organ function of control mice. Plasma alanine aminotransferase (ALT) (**a**) and lactate dehydrogenase (LDH) (**b**) levels in control groups (P, A, G, AG). (**c**) Representation of the differences in the distribution of cytokines (CKs) among septic/septic-treated group control groups (A, G, AG and P). The pro- and anti-inflammatory cytokines analyzed were interleukin-1β (IL-1β) (orange dots), IL-6 (grey dots), IL-10 (green dots), TGF-β1 (blue dots) and TNF-α (red dots). Each dot includes the above mentioned CKs of liver, kidney and lung. **(d**) Inducible nitric oxide synthase (iNOS) mRNA expression in the kidney, liver and lung from all control groups. (**e**) Representative images and quantification (as MPO intensity/number of nuclei) of myeloperoxidase staining (brown color) in spleen samples from control mice. For all analysis untreated healthy mice (P = PBS, N.=4), treated healthy mice (A = ampicillin, N.=4; G = Gas6, N.=4 and AG = ampicillin + Gas6, N.=4) were included. Values are represented as average ± SD.

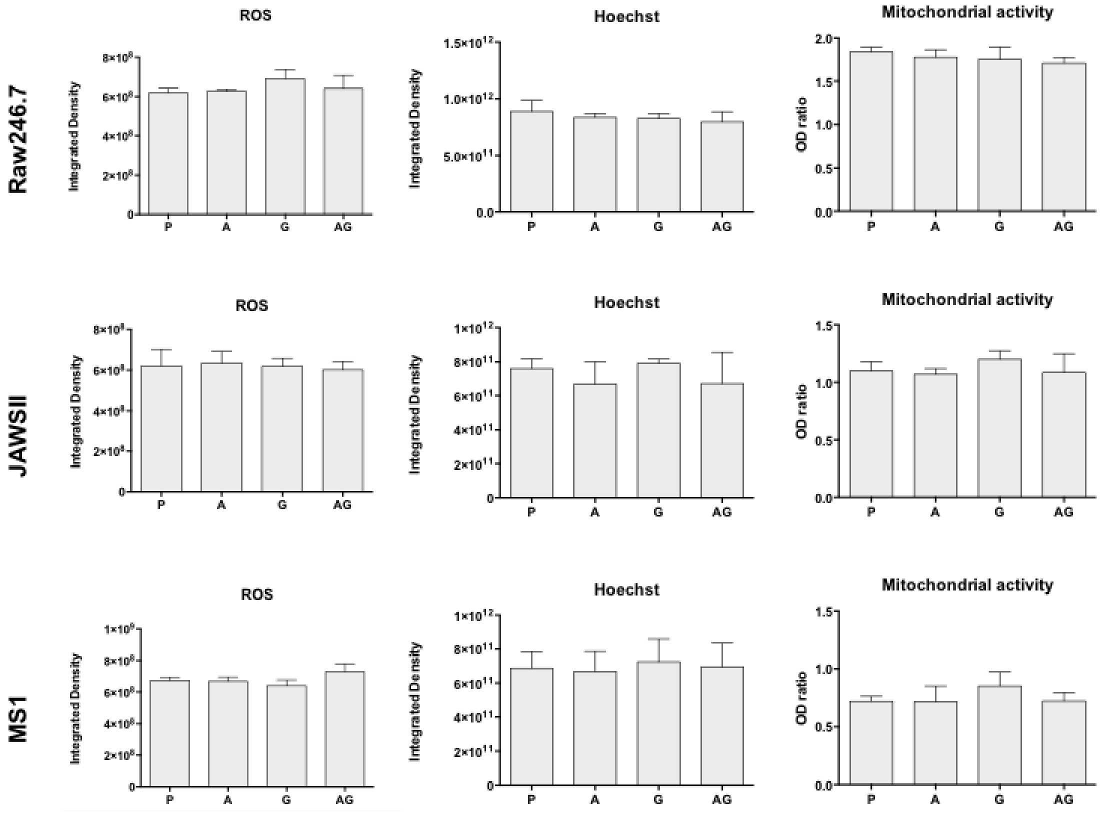

Figure S5. ***In vitro* control treatments.** Reactive oxygen species (ROS) production, cell viability and mitochondrial activity in Raw246.7, JAWSII and MS1 cells. For all analysis untreated healthy mice (P = PBS, N.=4), treated healthy mice (A = ampicillin, N.=4; G = Gas6, N.=4 and AG = ampicillin + Gas6, N.=4) were included. The histograms represent mean values ± SD.

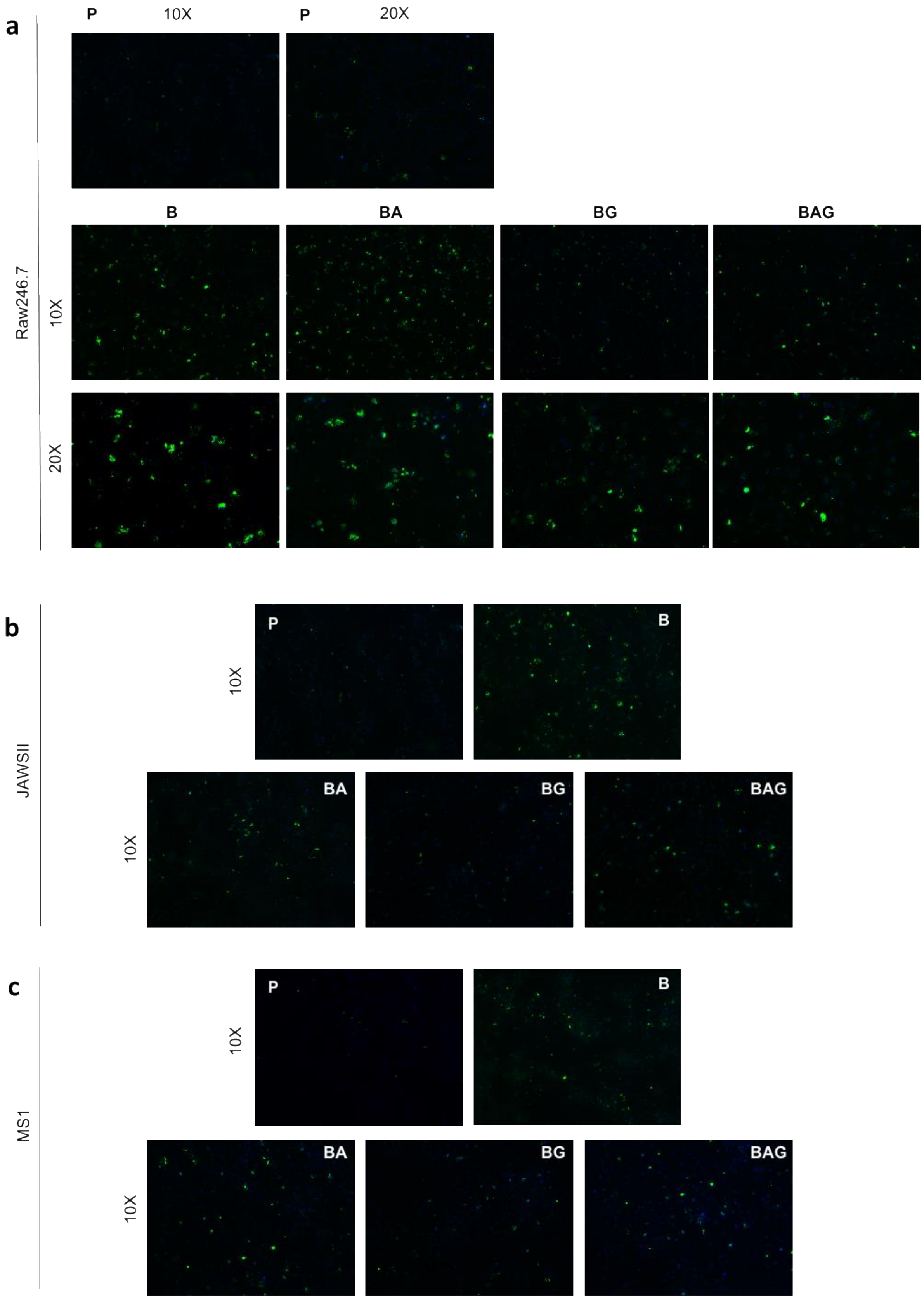

Figure S6. Representative fluorescent images of reactive oxygen species (ROS) investigated in Raw246.7 (a), JAWSII (b) and MS1 (c) cell lines treated as follow: P = PBS; B = PFA-inactivated (PFAi) bacteria; BA = PFAi bacteria + ampicillin; BG = PFAi bacteria + Gas6; BAG = PFAi bacteria+ ampicillin + Gas6.

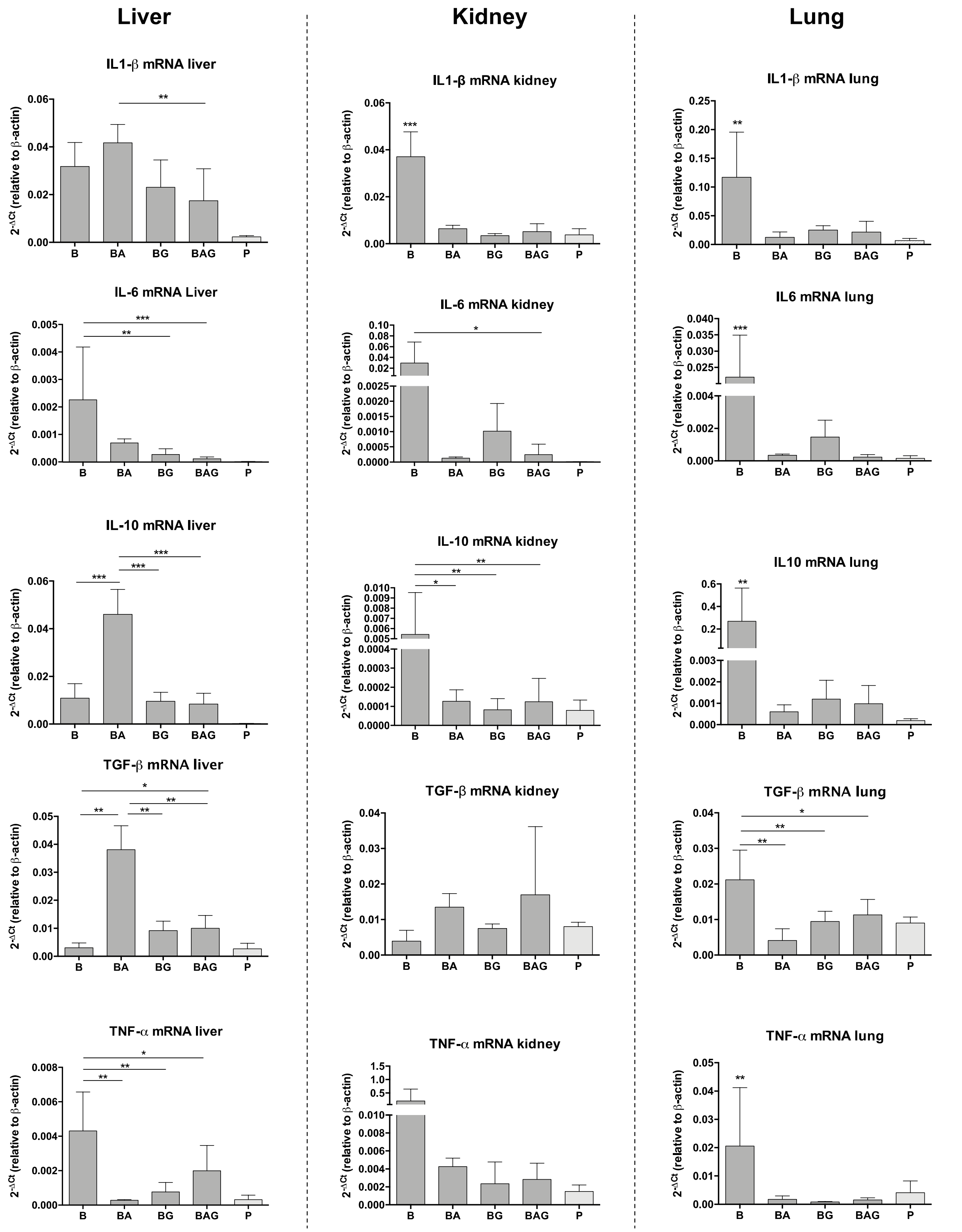

Figure S7. Relative expression ($2^{-\Delta Ct}$) of cytokines measured in liver, kidney and lung tissues. The pro- and anti-inflammatory cytokines analyzed were interleukin-1β (IL-1β), IL-6, IL-10, TGF-β1 and TNF-α. For all analysis septic mice (B = bacteria, septic mice N.=4), septic-treated mice (BA = bacteria + ampicillin, N.=4; BG = bacteria + Gas6, N.=4; BAG = bacteria + ampicillin + Gas6, N.=5) and control mice (P = PBS, N.=4) were included. Values are represented as average ± SD. * $P < 0.05$, ** $P < 0.01$, *** $P < 0.005$.
